# Effects of High Intensity Exercise on Oxidative Stress and Antioxidant Status in Untrained Humans: A Systematic Review

**DOI:** 10.3390/biology10121272

**Published:** 2021-12-04

**Authors:** Yining Lu, Huw D. Wiltshire, Julien S. Baker, Qiaojun Wang

**Affiliations:** 1 Cardiff School of Sport and Health Sciences, Cardiff Metropolitan University, Cardiff CF5 2YB, UK; st20184530@outlook.cardiffmet.ac.uk (Y.L.); hwiltshire@cardiffmet.ac.uk (H.D.W.); 2 Department of Sport, Physical Education and Health, Hong Kong Baptist University, Kowloon Tong, Hong Kong; jsbaker@hkbu.edu.hk; 3 Faculty of Sport Science, Ningbo University, Ningbo 315000, China

**Keywords:** high intensity exercise, oxidative stress, antioxidant, untrained

## Abstract

**Simple Summary:**

This systematic review aims to investigate the influence of HIE on oxidative stress and antioxidant status in untrained humans. Following the PRISMA selection process, 21 studies were finally included. A rigorous methodological quality assessment (MQA) and levels of evidence was applied. There was strong evidence that acute oxidative stress occurs at the cessation of HIE when compared to resting states. The HIE-induced oxidative stress is transient and recoverable due to the stimulated endogenous antioxidant system. While the responses of antioxidant systems are lagging and lasting, multiple measurement times are suggested. Higher fitness levels are associated with less oxidative stress and regular physical exercise appears to improve antioxidant capacity and provide health benefits.

**Abstract:**

Participation in exercise promotes health. High intensity exercise (HIE) has become increasingly popular among the general population, however, its effects on exercise-induced oxidative stress and antioxidant status in untrained humans is not clear. The aim of this systematic review was to investigate the influence of HIE on oxidative stress and antioxidant status in untrained humans. Web of Science, PubMed, MEDLINE, and Scopus were searched until March 2021. A methodological quality assessment valuation/estimation was additionally carried out in the final sample of studies. Following the PRISMA selection process, 21 studies were finally included. There was strong evidence that acute oxidative stress following the cessation of HIE exists when compared to resting states. The HIE-induced oxidative stress is transient and is most likely restored to normal levels within 24 h due to the stimulated endogenous antioxidant system whose response was lagging and lasting. Physically active humans had better antioxidant systems and suffered less oxidative stress after HIE. A physically active lifestyle was considered to enhance antioxidant capacity. For untrained humans, HIE with intensities above 70% VO_2_max are proposed for initial exercise levels based on the findings reported here.

## 1. Introduction

Free radicals are rogue molecules that damage cells. Denham Harman (1956) first discovered the active properties of free radicals and suggested the free radical theory of aging [1]. The theory proposed that the production of free radicals, such as reactive oxygen species (ROS), is inevitable during metabolism. ROS are active substances containing oxygen occurring in the human body or the natural environment. Normal metabolism in the body can produce ROS, which can initiate the formation of free radicals [2]. However, any uncontrolled production of reactive oxygen species can lead to oxidative damage to proteins, DNA, and lipids [3,4].

Antioxidants are substances that minimize the harmful effects of oxygen. These substances help trap and neutralize free radicals, thereby preventing damage on the human body. The antioxidant system in the human body consists of antioxidant metabolites and enzymes that impede the production of ROS by removing these active substances before they can cause damage to the important components of cells [5]. However, ROS are not always harmful, and physiologically, appropriate concentrations of ROS can promote immunity [6]. Therefore, the role of the body’s antioxidant system is not to remove ROS completely, but to control them at appropriate levels.

Physiologically, antioxidants and oxidants are in equilibrium. The body’s endogenous antioxidant defense system (non-enzymatic and enzymatic), under normal conditions, is effective against the potentially harmful effects of ROS [6,7]. When oxidizing substances increase, oxidative stress occurs. Increasing evidence shows that most health problems and diseases caused by aging are related to endogenous ROS production and oxidative stress [8]. It is widely believed that most age-related health problems, ranging from wrinkles, and including cardiovascular disease, cancer, and Alzheimer’s disease, are linked to excessive oxidative stress [9,10,11].

Many studies have shown that with the increase in oxygen consumption during exercise, the production of ROS increases [5,12]. When the ability of the antioxidant system is insufficient to counterbalance ROS production during exercise, oxidative stress occurs. Davies et al. (1982) used electron spin resonance (ESR) for the first time to directly confirm the significant increase of free radicals in muscles following exercise [13]. Over the past 40 years, various studies have emerged investigating the effects of oxidative stress induced by exercise.

Studies have also indicated that regular exercise can upregulate the body’s antioxidant system and increase its resistance to oxidative stress [14,15,16,17]. Regular exercise is beneficial to health, and it can reduce the risk of cancer, cardiovascular disease, diabetes, and other chronic diseases [2,8,18,19,20].

Despite this, Davies et al. (1982) and Jackson et al. (1985) used ESR to provide direct evidence that exercise may induce oxidative stress [13,21]. The elevation of biomarkers of oxidative damage in the blood and skeletal muscle also provides indirect evidence for oxidative stress induced by exercise. During exercise, skeletal muscle contractions produce free radicals, while increased oxygen consumption produces a large amount of ROS [22,23,24,25]. If the body’s antioxidant defense is insufficient, cells and tissues will suffer oxidative damage [26].

Physical exercise is a complex biological activity that constantly challenges the oxidation–antioxidant balance of the body (cells, tissues, organs, etc.) while maintaining biological balance [27]. The adjustment of exercise on oxidative stress can be acute or long-term. Acute adjustment is an incomplete adaptation that can easily lead to oxidative damage, so it is important to give the body sufficient rest following exercise to restore balance. The process of balancing—breaking the balance—restoring the balance helps improve the body’s ability to cope with oxidative stress. In fact, regular exercise can fundamentally upregulate the body’s endogenous antioxidant system [28]. Moderate aerobic exercise is often used to improve the body’s antioxidant capacity and reduce chronic diseases. At the same time, more and more studies suggest that high intensity exercise (HIE) may be more effective in promoting fitness and health than traditional continuous training [29,30,31].

Due to its efficient timesaving protocols and effectiveness, HIE has been the subject of more and more attention in recent years among athletes, bodybuilders, and individuals with chronic diseases. However, along with the increasing interest in HIE, there are questions that need consideration. One such question relates to the relationship between HIE and oxidative stress. This question may directly affect the arrangement of athletic training loads, the choice of fitness methods, and the safety of exercise for patients with chronic diseases.

To date, effects of HIE on oxidative stress in untrained humans are inconclusive. Therefore, this systematic review aims to systematically analyze effects of a single bout of HIE on oxidative stress markers and antioxidant status in untrained humans. A further aim was to investigate if long-term HIE can influence exercise-induced oxidative stress and upgrade the antioxidant system, and furthermore, to provide important information for physically inactive individuals to participant in HIE.

## 2. Methods

### 2.1. Data Sources and Searches

According to the Preferred Reporting Items for Systematic reviews and meta-analysis (PRISMA), a systematic literature search, limited to literature published in English and Chinese, was conducted in March 2021 using four electronic databases (PubMed, MEDLINE, Web of Science, SCOPUS). Following this process, the literature list obtained was then manually searched and the results were placed in Endnote (Endnote 20, Clarivate, Boston, MA, USA).

Search terms were limited to titles and abstracts and based on all possible combinations of the following keywords: high-intensity, interval, high-intensity interval, exhaustive, acute, training, exercise, exercised-induced, physical activity, oxidative stress, damage, oxidative damage.

### 2.2. Inclusion Criteria

#### 2.2.1. Type of Study

Studies involving high-intensity exercise protocols targeted at exploring the effects on oxidative stress markers and antioxidant status were included. Exercise protocols with a principal focus on high-intensity, high-intensity interval/intermittent, sprint, maximal, exhaustive, acute are considered as HIE in this review.

#### 2.2.2. Type of Participants

Studies conducted in healthy untrained humans were included. No gender constraints were applied to all participants without disability and obesity. Participants under 16 years old were not included. Studies that used HIE as a treatment for specific illnesses were excluded and so were animal studies. Participants were considered as untrained when participants were described as physically inactive, sedentary, non-athletes, recreationally active, and physically active.

#### 2.2.3. Type of Protocols

The inclusion criteria for studies in the review was as follows: (a) at least one bout of training/exercise was carried out; (b) oxidative stress markers were measured at baseline and post-training.

High intensity can be broadly defined as an intensity that is greater than that of exercise performed at a level corresponding to the anaerobic threshold. In this review, protocols were defined as high-intensity if: (a) the participant performed with an “all-out” effort [32]; (b) protocols were described as “maximal”, “sprint”, or “high”; (c) the intensity was ≥70% maximal oxygen uptake (VO_2_max); (d) the participants’ heart rates were ≥70% of their maximal heart rate. (e) There were no restrictions applied regarding the mode and the duration of the protocol.

#### 2.2.4. Type of Outcomes

Outcomes included oxidative markers (directly detected by electron spin resonance (ESR) and indirectly measured by malondialdehyde (MDA) and thiobarbituric acid reactive substances (TBARS)), antioxidant enzyme activities (superoxide dismutase (SOD), glutathione peroxidase (GPX), catalase (CAT) and glutathione (GSH)), and total antioxidant capacity (TAC).

Articles that satisfied the above criteria were included in the review. Meanwhile, articles were excluded if (a) they were published after March 2021; (b) full text of the articles was not found; (c) articles were not written in English or Chinese; (d) studies that used a different intervention (e.g., drugs or diet) that may have impacted on oxidative stress were excluded. When the same data were presented in multiple publications, the first published study was used for the review and analysis.

### 2.3. Identification of Eligible Studies

Eligible studies were empirical studies conducted in untrained humans that measured oxidative markers and antioxidant enzyme activities after a single bout of HIE or a long-term HIE protocol. Two authors (Yining Lu and Qiaojun Wang) were responsible for retrieving selected articles from four databases and applying inclusion and exclusion criteria to determine eligible studies. The articles were then carefully read and evaluated by a further two independent authors (Huw Wiltshire and Julien Baker) to determine whether they should be included.

### 2.4. Quality Assessment

The results were analyzed using methodological quality assessment (MQA) according to the revised Downs & Black Quality Index (1998) (Appendix A) [33]. The MQA was implemented by two authors (Yining Lu and Qiaojun Wang) and were proofread by the other authors. Finally, a consultation session was arranged to reconcile any differences. The revised edition contained a total of 10 questions; 5 of the questions assessed report quality, 4 assessed internal validity, and 1 assessed power. A “yes” or “no” for each question was recorded as a 1 or 0, respectively. The total score was 10. Studies were defined as high quality if they scored an overall score of 7 or higher. Studies were defined as low quality if they received a total score of 5 or 6, and studies were defined as very low quality when they obtained a score under 4 [34].

### 2.5. Level of Evidence

The levels of evidence were divided into three levels. Evidence was strong when three or more high-quality studies indicated consistent findings. The evidence was considered moderate when two high-quality studies showed consistent results. The evidence was limited when it was based on low-quality studies or a single study [34].

### 2.6. Data Extraction

The data included in the study were extracted in several structured table formats covering the following topics: sociodemographic characteristics of participants (age, gender, weight, body mass index, maximal oxygen uptake, diet, lifestyle, socio-economic level, tobacco, and alcohol), exercise protocol (specified as modality, type of protocol, No. of bouts, duration of bouts, duration of protocols, work/rest ratio, intensity), training protocols (specified as duration, frequency, No. of bouts, duration of bouts, intensity, duration of recovery), selected biomarkers, and findings. All studies measured the baseline status of the subjects. Some studies measured oxidative damage and antioxidant status at only one time-point (TP), mostly at the cessation of exercise, while others included multiple post-exercise measures following exercise completion.

## 3. Results

### 3.1. Search Results

**Figure 1:**
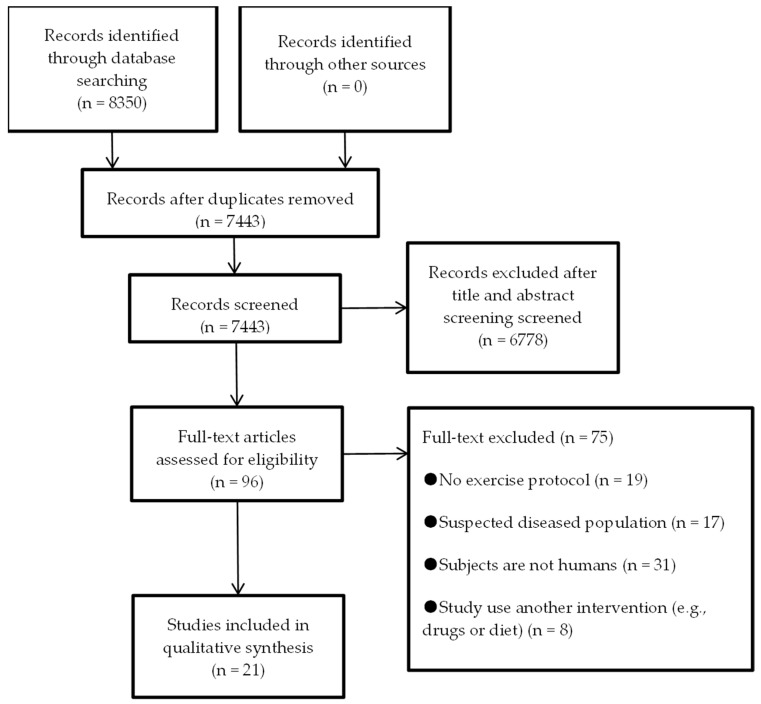
PRISMA flow diagram displaying the selection process.

The selection process and the number of articles identified at each step are shown in Figure 1. Eight thousand three hundred and fifty records (8350) were retrieved in the initial database search. Nine hundred and seven (907) duplicates were removed. After title and abstract screening, ninety-six (96) records were reserved for eligibility assessment. Seventy-three (75) articles were excluded after full-text examination (excluded reasons detailed in Figure 1). The most common reason for exclusion was that the participants were not human. Finally, twenty-one (21) were included in the present review.

### 3.2. Methodological Quality Assessment

The MQA scoring results of the selected 21 manuscripts are shown in Table 1. The total quality scores of the papers are shown as a percentage value in the last column. The quality of manuscripts ranged from 60% to 80%, and the average quality index was 69%. Twelve studies were high-quality (score ≥ 7), nine studies were low-quality (5 ≤ score ≤ 6), and no study was defined with a very low quality (score ≤ 4).

All manuscripts specified objectives (21/21), characteristics of the participants (21/21), findings (21/21), use of statistical tests (21/21), and a significance level of *p* < 0.05 (21/21). In addition, 11 of the 21 studies explicitly mentioned the intervention, and 8 of the 21 studies were randomized. Finally, none of studies reported adverse events because of the interventions.

### 3.3. Type of Studies

Details of exercise testing and training regimes of the included studies are outlined in Table 2. Exercise testing involved exercise protocols used for analysis of the acute changes in oxidative stress and antioxidant status, while training were protocols used to investigate the effect of long-term exercise on oxidative stress and antioxidant capacity. Thus, 15/21 studies investigated acute oxidative response following a single bout of HIE at TP0 (Table 3 details corresponding TPs and findings for each study); 13/21 studies included multiple post-exercise measures after exercise; 3/21 studies compared HIE-induced oxidative stress and antioxidant status pre and post high intensity training; 2/21 studies measured oxidative damage between untrained participants and others with different physical activity characteristics; 8/21 studies compare oxidative stress following different types of HIE.

### 3.4. Participants of Selected Studies

Table 4 outlines the sociodemographic characteristics of participants. Participants mean age in selected studies ranged from 17.3 to 65.1 years old. Participants body mass index ranged from 21.5 to 27.6. VO_2_max of participants ranged from 31.7 to 49.7 mL/kg/min. Sixty studies reported no medications and antioxidant dietary supplement prior and throughout the test [35,36,37,38,40,41,42,43,44,45,47,50,51,52,54,55]. Eight studies described participants lifestyle as physically active [36,37,38,42,44,45,51,55], one study described as physically inactive [35], three studies decried as no regular physical activity [40,43,49], and six studies used sedentary participants [39,41,46,50,52,53]. Two studies included groups of trained participants [40,41]; one used handball athletes with regular training [40] and the other used amateur runners [41]. Nine studies used non-smoking participants [40,42,47,50,51,52,53,54,55], and two studies reported participants refrained from tobacco in the last 6 months [44,45]. Finally, eight studies reported participants with no alcohol at least 24 h prior to the test [40,41,44,45,47,50,51,54].

### 3.5. Oxidative Stress Markers

Seven oxidative stress markers were analyzed, these included: thiobarbituric acid reactive substances (TBARS) (9/21), malondialdehyde (MDA) (8/21), glutathione (GSH) (4/21), glutathione peroxidase (GPX) (8/21), superoxide dismutase (SOD) (8/21), catalase (CAT) (10/21), and total antioxidant capacity (TAC) (8/21) (Table 5).

### 3.6. Exercise Modes

In terms of exercise modality, cycling on ergometers were the most common HIE used, three used treadmills [39,41,52], and one used a shuttle run [48]. In terms of exercise intensity, there were nine studies that included incremental exercise [37,39,40,41,42,46,49,50,53], six performed a single bout of exhaustive/maximal exercises [35,36,37,44,48,55], six implemented interval exercises [38,43,45,47,51,54], and five conducted continuous exercise [35,37,47,52,54]. The duration of a single high intensity exercise bout ranged from 20 s to 5 min.

### 3.7. Levels of Evidence

Conclusive strong evidence was obtained in the selected high-quality samples. In terms of acute oxidative stress assessed immediately following a bout of HIE, among these studies, oxidative stress was significantly increased in five high-quality studies [35,36,39,48,49], and antioxidant status was significantly stimulated in seven high quality studies [35,37,39,41,47,48,51]. Moderate evidence on significantly decreased oxidative damage was found in two high quality studies [40,51]. However, the evidence that antioxidant status was not affected immediately after HIE is also strong due to three high-quality studies [40,49,54].

In relation to the effects of different protocols and measurement time on oxidative stress, we observed the following results:

#### 3.7.1. Acute Effect of Oxidative Stress and Antioxidant Status after HIE

Seventeen studies assessed the acute response of oxidative stress immediately following HIE (TP0 and TP1). Among them, nine studies reported significantly increased acute oxidative damage immediately post HIE [35,36,38,39,42,43,44,48,49]. Four studies reported significant acute oxidative damage following a maximal exercise [35,36,44,48]. Three studies used incremental protocols [39,42,49] and another two used intermittent protocols [38,43]. On the contrary, only two studies reported significantly decreased acute oxidative damage [40,51]. One study used incremental exercise [40], and the other study adopted an interval protocol. Six studies did not observe significant changes [41,46,47,50,52,53]. Three of them used incremental protocols [41,46,53], one of them executed high intensity interval exercise [50], and one of them used high-intensity continuous exercise [52]. The remaining one compared interval exercise with continuous exercise performed at a high intensity, indicating no between-group differences regarding acute oxidative responses [47]. It was worth noting that one study also used electron spin resonance (ESR) to directly test the production of lipid free radicals to study oxidative stress responses to maximal exercise [44]. The authors found that lipid free radicals increased significantly after exercise, while plasma TBARS concentrations did not increase.

For antioxidant status immediately after HIE, data were available from eighteen studies. Twelve studies reported the alterations of redox homeostasis. Most studies (9/18) indicated elevated antioxidant enzyme activities [35,37,38,39,42,43,47,48,50]. There were four studies that used interval exercise [38,43,47,50]. Three studies used incremental protocols [37,39,42] and three studies used maximal exercises [35,37,48]. Two performed high-intensity continuous exercises [37,47] and only one applied a combined protocol, consisting of maximal exercise followed by a moderate continuous exercise [35]. Among them, four studies investigated antioxidative responses between different types of protocols [35,37,47,50]. In contrast, three studies indicated decreased antioxidant activities [41,44,51]. Incremental [41], interval [51], and maximal [44] protocols were used. Six studies reported no changes in antioxidant activities immediately after HIE [40,46,49,53,54,55]. Four of them performed incremental protocols [40,46,49,53]. One used maximal exercise [55] and the remaining study compared antioxidant changes between high-intensity interval and high-intensity continuous exercises [54].

#### 3.7.2. Time of Measurement Effects of Oxidative Stress and Antioxidant Status after HIE

Eleven studies used multiple post-exercise measures on oxidative markers after exercise. Eight studies observed increased oxidative damage following HIE [35,36,38,42,43,46,52,53]. Among them, increased oxidative damage was observed from TP0 (0 min) to TP9 (48 h) following HIE, with peak values at various time points. Peak oxidative damage occurred at TP1 (5 min) in two studies [46,53]. One study reported peak values at TP6 (2 h) [52]. Another study observed the highest oxidative damage at TP8 (24 h) [38]. No conclusive peak value could be found in four studies [35,36,42,43]. Generally, oxidative damage returned to baseline within 24 h after HIE in most studies (5/8) [36,42,43,52,53]. Only one study reported increased damage after 24 h [38]. No recovery data could be recorded in the other two studies due to the limited measurement time [35,46].

Two studies reported decreased oxidative stress markers from TP0 (0 min) to TP6 (2 h) [44,51]. Only one study indicated no change in oxidative stress at any measurement time from TP0 (0 min) to TP10 (72 h) [47].

For endogenous redox status, data was analyzed from eleven studies [35,38,42,43,44,46,47,51,53,54,55]. All studies reported significant changes in antioxidants from TP0 (0 min) to TP4 (30 min). Seven studies indicated early alterations within 5 min after HIE [35,42,43,44,46,47,51], while two studies did not observe significant changes until 30 min [38,54]. Another two studies indicated altered antioxidant status from 10 min to 15 min following HIE [51,53].

## 4. Discussion

This systematic review aimed to investigate the effects of HIE on oxidative stress and antioxidant capacity in untrained adults. The results suggest that HIE induces oxidative stress compared to a resting state. Regardless of whether HIE is performed on treadmills, cycle ergometers, or other exercise types, if the duration is more than 30 s and VO_2_max reaches 70% or more, the balance of oxidative and antioxidant systems in the body will be disrupted, leading to oxidative stress and cellular damage. The results also show that regular exercise, sufficient recovery, and young age increased protection against exercise-induced oxidative damage; however, further studies are needed to confirm and explore this finding.

HIE can interfere with the balance between oxidation and anti-oxidation systems in the body. During HIE or during a short period following HIE, the production of ROS significantly increases, which is related to the sharp increase in oxygen consumption, activation of inflammatory cells, and contraction of muscle. However, the endogenous antioxidant capacity was simultaneously elevated.

HIE can be either aerobic or anaerobic exercise, or a combination of both. Incremental exercise, maximal exercise, intermittent exercise and high intensity continuous exercise at 70% VO_2_max or above were the common HIE methodologies used in untrained adults.

Studies that refer to different types of HIE report the almost conclusive finding that (1) HIE induces oxidative stress, (2) HIE-induced oxidative stress is transient, (3) antioxidant capacity is also activated after HIE, (4) regular exercise enhanced the antioxidant defense mechanisms, (5) HIE-induced oxidative stress is related to individual subject characteristics.

### 4.1. HIE Induces Oxidative Stress

HIE induces oxidative stress, regardless of the mode of exercise. In this review, cycling and running were the more common exercises used. A cycling exercise to exhaustion can induce oxidative stress [35,36,37,38,42,43,46,47,49,50,53,54,55] and a running exercise to exhaustion has a similar effect [39,41,48,52]. This has been evidenced by a recent study [56], in which oxidative stress was assessed using two different HIE modalities: running and cycling. The study concluded that both cycling and running induce oxidative stress, even though TAC recovers faster among runners.

Furthermore, the intensity of HIE can also influence oxidative stress. Using an incremental intensity, Parker et al. (2014) observed significant oxidative stress at intensities of 70% VO_2_max or above with increasing oxidative stress accompanied by increased exercise intensity [50]. This has also been reported by Fogarty et al. (2011), who conducted three aerobic exercises at 40%, 70%, and 100% of VO_2_max [57]. Oxidative damage to DNA was increased at 70% and 100% VO_2_max and the extent of damage was positively related to the intensity. Similarly, during a 30 s maximal cycling test, the selection of resistive forces (TBM or FFM) may induce different metabolic responses for oxidative stress [36]. This study indicated that a FFM protocol was metabolically more efficient compared to the TBM protocol and produced less oxidative stress and muscle damage during exercise. It can be concluded that when the exercise intensity is higher than 70% VO_2_max, significant oxidative stress occurs, and the extent of oxidative damage is positively related to the intensity. This finding is consistent with the recent work of Tryfidou et al. (2020), who reported DNA oxidative stress damage increased after exercise with intensities higher than 75% [58].

The duration of HIE can also be one of the most important factors for exercise-induced oxidative stress. Using data from this review, we observed that for a single high-intensity exercise bout, oxidative stress occurs when the exercise duration is more than 30 s. The most common HIE protocol consists of several 30 s “all-out” bouts separated by recovery. As such, we believe that most HIE protocols will induce oxidative stress. However, a single bout duration shorter than 30 s was proven to be associated with less oxidative stress [59]. With the same modality and intensity. Cipryan (2017) performed 3 HIE protocols with a total of 12 min exercise [59]. The durations were 15 s, 30 s, or 60 s, respectively, and the work/rest ratio was 1. The authors observed an immediate increase in oxidative stress markers in all three protocols. Among them, oxidative stress in 30/30 protocol showed the smallest increases, while the TAC in 15/15 protocol demonstrated the largest increase. However, further studies are needed to investigate oxidative damage following exercises performed at the same intensity but using different modalities (continuous or intermittent).

### 4.2. HIE-Induced Oxidative Stress Is Transient

Using data from this review, we conclude that there is a significant increase in oxidative stress markers following exercise at TP0 (0 h) when compared to rest. To investigate oxidative damage after exercise at multiple TP, data were available from 13 studies (details in Table 3). This review demonstrates the acute effect on oxidative stress following high-intensity exercise often occurs within 5 min at the end of the exercise, remaining elevated within 30 min following exercise. Moreover, several studies reported that oxidative stress peaked at 5 min post exercise. Most of the increased oxidative damage returned to basal level within 24 h following exercise cessation [36,42,43,52,53]. Researchers observed that the greatest oxidative stress occurred in healthy subjects 5 min post exercise and then recovered gradually, with different markers recovering at different rates [46,53].

Basically, responses of the antioxidant enzymes to oxidative stress are lagging and lasting. Significant changes of SOD, CAT, and TAC were observed at 15 min after exercise [55]. SOD was increased continuously until 3 h of the end of exercise [43]. Finkler et al. (2016) also indicated that TBARS concentrations increased immediately, whereas the activation of CAT appears to be small and continues to increase during the recovery period [42]. Similarly, TAC is significantly elevated from rest to post exercise and remained above pre-exercise levels for 24 h [59,60,61]. This agrees with the previous study. Farney et al. (2012) reported an absence of oxidative stress in trained men following HIE [62]. One study reported significant changes in antioxidant activities were found 20 min post exercise [63]. Therefore, stimulated antioxidant defense could be observed or not at the cessation of HIE.

### 4.3. The Antioxidant Capacity Is also Activated after HIE

The degree of oxidative stress depends on the balance between the generation of ROS and the effectiveness of the antioxidant defense system. Studies have shown that the antioxidant defense system in the body was rapidly activated after HIE [37,44,47,48,50,64,65]. Fisher et al. (2011) also found that the absence of lymphocyte cell viability decreases after HIE exercise and was due to the increased activity of antioxidant enzymes in lymphocytes [43]. Many studies have also shown a significant increase in antioxidant activity after HIE compared to baseline values [38,43,46,59,60,61,66,67,68]. In contrast, several studies found that antioxidant enzyme activities did not increase but decreased after HIE. Others, Falone et al. (2010) and Kröpfl et al. (2021), also found that some endogenous antioxidants did not change after exercise [41,56]. This supported one of our observations that antioxidative status did not change after HIE. Such results demonstrate that oxidative stress caused by HIE had been rapidly neutralized by the antioxidant system, and that there was no significant change in antioxidant markers.

Whether moderate oxidative stress, which stimulates the antioxidative system temporarily and appropriately, contributes to the improvement of antioxidant levels in the body deserves further investigation.

### 4.4. Regular Exercise Enhanced the Antioxidant Defense Mechanisms

Individuals who are physically active have better antioxidant systems and can respond to oxidative stress induced by HIE more quickly than sedentary individuals. In the resting state, physically active people have higher baseline values of TAC, and lower TBARS [40,41,60,65]. After exercise, TAC showed the largest changes, indicating that regular exercise can improve the activity of the antioxidant system and reduce exercise-induced oxidative damage. It also shows that under the same HIE protocol, the higher the fitness levels of individuals, the less obvious the oxidative stress increases should be after exercise. In addition, physically active individuals have stronger ability to counter oxidative stress, affecting the recovery time of oxidative indicators, such as TBARS, MDA, and TAC, which did not change immediately after exercise [22,64,69,70,71]. Even in the study of Groussard et al. (2003), a sharp post-exercise decrease was observed in plasma TBARS and MDA levels in university physical education students [44]. This is more likely to happen in individuals who are physically active.

Compared with a single bout of HIE, however, short-term HIE leads to different conclusions in relation to the effects on oxidative stress. Faruk et al. (2013) conducted a 10-day HIE protocol and concluded that HIE could improve the oxidative stress of participants [72]. On the contrary, a HIE protocol lasting 3 weeks with training frequency of 3 times weekly and the total training time over 2 h was proven to reduce oxidative stress and to upregulate the antioxidant system [38]. In the study of Fisher et al. (2011), three HIE protocols were completed with 2 days’ recovery between each session [43]. The authors found that oxidative stress occurred on the first and second session but did not significantly increase in the third session. Similar findings were reported by Miyazaki et al. (2001) [49]. Furthermore, Falone et al. (2010) specified that long-term regular and moderate aerobic physical activity can increase antioxidant capacity [41]. Similar findings were provided by Hajizadeh et al. (2017) [45]. After a long period of moderate intensity continuous training, high-intensity continuous training and high-intensity interval training, the authors indicated three types of interventions attenuated oxidative stress with different kinetics and moderate intensity continuous training was superior in the promotion of antioxidant capacity.

Using the evidence from the studies reviewed, we can infer that a single session of HIE can induce oxidative stress; however, a HIE protocol lasting a longer period will attenuate oxidative stress. This statement agrees with the findings that regular HIE could keep oxidative DNA damage at a lower level for a long period [73] and consecutive days of high-intensity exercise improved endogenous antioxidant capacity and reduced exercise-induced oxidative stress [74]. However, further studies are needed to explore the efficiency of long-term high intensity exercise and moderate intensity exercise on antioxidant capacity.

### 4.5. HIE-Induced Oxidative Stress Is Related to Individual Characteristics

In terms of gender, oxidative stress in both male and female individuals changed following HIE [72]. In the study of Jammes et al. (2004), the maximum increase in plasma TBARS after exercise was slightly higher in men than in women, while this difference was almost negligible [46]. Furthermore, Wiecek et al. (2018) indicated no differences concerning changes in antioxidant activity post HIE between males and females, while males represented higher level of baseline antioxidant activity [55]. However, Steinberg et al. (2007) and Jammes et al. (2004) observed that maximal increase in TBARS was positively related to VO_2_max [46,53]. From these studies, we can conclude that high-intensity exercise-induced oxidative stress is not related to gender. Since limited studies focused on women, more studies exploring oxidative stress using different gender participants are needed.

Generally, aging is related to a decline in antioxidant capacity and aged populations are more susceptible to oxidative stress. Bouzid et al. (2014) compared changes in oxidative stress with aging populations at rest and post HIE [39]. There was no difference in oxidative markers between young and elderly groups at rest and antioxidant activities only increased in the young group post exercise. Furthermore, Boisseau et al. (2000) found that post-pubertal boys have greater muscle mass, higher mitochondrial respiration, and greater oxygen uptake during exercise [75]. This resulted in greater ROS production and subsequent oxidative stress because of puberty. At the same time, post-pubertal boys had higher antioxidant capacity. Higher baseline value of TBARS, CAT, and SOD were observed in post-pubertal populations, and more significant changes in these markers after exercise were found. Therefore, post-pubertal populations were considered to have a stronger ability to counter oxidative stress [67].

The findings from cross-sectional studies are limited. However, longitudinal studies are needed to demonstrate the response of aging and fitness levels in relation to oxidative stress and antioxidant capacity.

## 5. Limitations and Strengthens

There are several limitations in the present review. Firstly, the heterogeneity among selected studies is considerable (I^2^ > 75%). This may be due to the exercise protocols using different characteristics, making it difficult to draw a more precise conclusions regarding exercise type, duration, and intensity. Second, although most studies reported no medications and antioxidant dietary supplement before and during the test, they did not analyze daily diet, which could have an impact on the results. Finally, this review chose to focus solely on studies that have reported oxidative stress assayed from blood concentrations as this represents the most frequently used indicator. We acknowledge that oxidative damage can also be detected directly by ESR or indirectly in urine and muscle indicators. Furthermore, this is the first systematic review investigating the influence of HIE with untrained humans and MQA was executed rigorously through all the selected studies.

## 6. Practical Applications

- High-intensity exercise-induced oxidative stress is acute and recoverable, and in young healthy untrained humans, oxidative stress after a single bout of high intensity exercise will not be elevated to dangerous levels.
- Higher physical fitness level is associated with shorter time to recovery from the exercises induced oxidative stress.
- Higher intensity is related to higher exercise-induced oxidative stress, and 70% VO_2_max with sufficient recovery is a better exercise mode for untrained humans to initiate high-intensity exercise.
- Establishing a standardized high-intensity exercise protocol in order to specifically investigate oxidative responses post exercise will help provide a better knowledge in this area.

## 7. Conclusions

This systematic review demonstrates that an increase in oxidative damage occurs following a HIE bout. The data further demonstrate that oxidative stress was positively associated with increases in exercise intensity, while benefits were observed in studies using more than one HIE session. Although oxidative stress occurs after HIE, this is not a negative outcome per se. Such exercise-induced oxidative stress is transient and most likely recovers within 24 h, or even sooner, as studies using multiple TP measurements suggest. Such acute oxidative stress does not have any long-term harmful health outcomes. On the contrary, short-term oxidative stress can stimulate the body’s antioxidant system, which in the long term will improve the body’s antioxidant capacity and have a positive effect on health promotion.

The exercise modality during HIE is not related to oxidative stress, but the intensity and duration of HIE are closely related to increases in oxidative stress. It is generally believed that the greater the intensity and the longer the duration of HIE, the more intense the oxidative stress would be. At the same time, the degree of oxidative stress is also related to the individual’s exercise habits, and individual fitness levels and age. Gender also appears to be associated with resting levels of antioxidant capacity, with greater values recorded in males. Individuals who are physically active appear to have greater antioxidant capacities. From the findings of this review, we can conclude that HIE can be an alternative for untrained humans to improve antioxidant capacity and promote health.

However, the combination of frequency, intensity, and duration of HIE protocols needs to consider individual characteristics comprehensively when prescribing individual training programs, since induced oxidative stress level responses are not identical or specific between individuals.

## Figures and Tables

**Table 1:** Methodological quality assessment.

Reference	Q1	Q2	Q3	Q4	Q5	Q6	Q7	Q8	Q9	Q10	Total
Ammar et al., 2020 [35]	1	1	1	1	0	0	1	1	1	1	80%
Baker et al., 2004 [36]	1	1	1	1	0	0	1	1	1	1	80%
Berzosa er al., 2011 [37]	1	1	1	1	0	0	1	1	1	1	80%
Bogdanis et al., 2013 [38]	1	1	1	1	0	0	1	0	0	1	60%
Bouzid et al., 2014 [39]	1	1	1	1	0	0	1	1	0	1	70%
Djordjevic et al., 2012 [40]	1	1	1	1	0	0	1	1	0	1	70%
Falone et al., 2010 [41]	1	1	1	1	0	0	1	1	0	1	70%
Finkler et al., 2016 [42]	1	1	1	1	0	0	1	0	0	1	60%
Fisher et al., 2011 [43]	1	1	1	1	0	0	1	0	0	1	60%
Groussard et al., 2003 [44]	1	1	1	1	0	0	1	0	0	1	60%
Hajizadeh et al., 2017 [45]	1	1	1	1	0	0	1	1	1	1	80%
Jammes et al., 2004 [46]	1	1	1	1	0	0	1	0	0	1	60%
Jamurtas et al., 2018 [47]	1	1	1	1	0	0	1	1	1	1	80%
Kyparos et al., 2007 [48]	1	1	1	1	0	0	1	0	1	1	70%
Miyazaki et al., 2001 [49]	1	1	1	1	0	0	1	1	0	1	70%
Parker et al., 2014 [50]	1	1	1	1	0	0	1	0	0	1	60%
Parker et al., 2018 [51]	1	1	1	1	0	0	1	1	1	1	80%
Seifi-Skishahr et al., 2008 [52]	1	1	1	1	0	0	1	0	0	1	60%
Steinberg et al., 2007 [53]	1	1	1	1	0	0	1	0	0	1	60%
Wadley et al., 2016 [54]	1	1	1	1	0	0	1	1	1	1	80%
Wiecek et al., 2018 [55]	1	1	1	1	0	0	1	0	0	1	60%
	Reporting					Internal validity—bias		Internal validity—confounding		Power	Average
TOTAL/21	21	21	21	21	0	0	21	11	8	21	69%

**Table 2:** Details of exercise testing and training of the included studies.

Reference	Exercise Testing							Training
	Modality	Type of Protocol	No. of Bouts	Duration of Bouts	Duration of Protocol	W/R Ratio	Intensity (W/R)	Duration, Frequency, (No. of Bouts) × (Duration of Bout/Intensity)/Duration of Recovery
Ammar et al., 2020 [35] (anerobic)	Cycling	Maximal	1	30 s	30 s		All-out	
Ammar et al., 2020 [35] (combined)	Cycling	Combined (maximal and moderate intensity continuous)	1	30 s + 30 min	30 s + 30 min		All-out + 60% MAP	
Baker et al., 2004 [36] (TBM)	Cycling	Maximal	1	30 s	30 s		All-out	
Baker et al., 2004 [36] (FFM)	Cycling	Maximal	1	30 s	30 s		All-out	
Berzosa er al., 2011 [37] (incremental)	Cycling	Incremental	1				Incremental intensity to exhaustion	
Berzosa er al., 2011 [37] (100% VO_2_max)	Cycling	Maximal	1				100% VO_2_max to exhaustion	
Berzosa er al., 2011 [37] (70% VO_2_max)	Cycling	High-intensity continuous	1	30 min	30 min		70% VO_2_max	
Bogdanis et al., 2013 [38] (pre-training)	Cycling	Sprint interval	4	30 s	14 min	1/8	All-out/Active recovery	3 weeks, 3 sessions/week, (4–6) × (30 s/all-out)/4 min
Bogdanis et al., 2013 [38] (post-training)	Cycling	Sprint interval	4	30 s	14 min	1/8	All-out/Active recovery	
Bouzid et al., 2014 [39] (young)	Treadmill	Incremental	1				Incremental intensity to exhaustion	
Bouzid et al., 2014 [39] (old)	Treadmill	Incremental	1				Incremental intensity to exhaustion	
Djordjevic et al., 2012 [40] (athletes)	Cycling	Incremental	1				Incremental intensity to exhaustion	
Djordjevic et al., 2012 [40] (non-athletes)	Cycling	Incremental	1				Incremental intensity to exhaustion	
Falone et al., 2010 [41] (amateur runner)	Treadmill	Incremental	1				Incremental intensity to exhaustion	
Falone et al., 2010 [41] (untrained)	Treadmill	Incremental	1				Incremental intensity to exhaustion	
Finkler et al., 2016 [42]	Cycling	Incremental	1				Incremental intensity to exhaustion	
Fisher et al., 2011 [43] (first)	Cycling	High-intensity interval	4	30 s	14 min	1/8	90% Max-AP/15% Max-AP	
Fisher et al., 2011 [43] (second)	Cycling	High-intensity interval	4	30 s	14 min	1/8	90% Max-AP/15% Max-AP	
Fisher et al., 2011 [43] (third)	Cycling	High-intensity interval	4	30 s	14 min	1/8	90% Max-AP/15% Max-AP	
Groussard et al., 2003 [44]	Cycling	Maximal	1	30 s	30 s		All-out	
Hajizadeh et al., 2017 [45] (HICE)	Treadmill	High-intensity continuous	4	10 min	49 min	10/3	70–75% VO_2_max/50–60% VO_2_max for the first 12 weeks; 75–85% VO_2_max/50–60% VO_2_max for the final 12 weeks	24 weeks, 3 sessions/week, 4 × [10 min/(70–75%) − (75–85%)VO_2_max]/3 min
Hajizadeh et al., 2017 [45] (HIIE)	Treadmill	High-intensity interval	10	1 min	19 min	1/1	75–85% VO_2_max/45–50% VO_2_max for the first 12 weeks; 85–95% VO_2_max/45–50% VO_2_max for the final 12 weeks	24 weeks, 3 sessions/week, 10 × [1 min/(75–85%) − (85–95%)VO_2_max]/1 min
Jammes et al., 2004 [46]	Cycling	Incremental	1				Incremental intensity to VO_2_max	
Jamurtas et al., 2018 [47] (HIIE)	Cycling	Sprint interval	4	30 s	14 min	1/8	All-out	
Jamurtas et al., 2018 [47] (HICE)	Cycling	High-intensity continuous	1	30 min	30 min		70% VO_2_max	
Kyparos et al., 2007 [48]	Shuttle run	Maximal	1				All-out	
Miyazaki et al., 2001 [49] (pre-training)	Cycling	Incremental	1				Incremental intensity to exhaustion	12 weeks, 5 sessions/week, 1 × (60 min/80% VO_2_max)
Miyazaki et al., 2001 [49] (post-training)	Cycling	Incremental	1				Incremental intensity to exhaustion	
Parker et al., 2014 [50] (70% VO_2_max)	Cycling	High-intensity interval	1	5 min	5 min	5/12	70% VO_2_max/passive seated rest	
Parker et al., 2014 [50] (85% VO_2_max)	Cycling	High-intensity interval	1	5 min	5 min	5/12	85% VO_2_max/passive seated rest	
Parker et al., 2014 [50] (100% VO_2_max)	Cycling	High-intensity interval	1	5 min	5 min	5/12	100% VO_2_max/passive seated rest	
Parker et al., 2018 [51] (HIIE)	Cycling	High-intensity interval	5	4 min	24 min	4/1	75%Wmax	
Parker et al., 2018 [51] (SIE)	Cycling	Sprint interval	4	30 s	15.5 min	1/9	All-out	
Seifi-Skishahr et al., 2008 [52]	Treadmill	High-intensity continuous	1	30 min	30 min		75% VO_2_max	
Steinberg et al., 2007 [53]	Cycling	Incremental	1				Incremental intensity to exhaustion	
Wadley et al., 2016 [54] (LV-HIIE)	Cycling	High-intensity interval	10	1 min	19 min	1/1	90% VO_2_max	
Wadley et al., 2016 [54] (HICE)	Cycling	High-intensity continuous	1	20 min	20 min		80% VO_2_max	
Wiecek et al., 2018 [55]	Cycling	Maximal	1	20 s	20 s		All-out	

Note: FFM, fat-free mass; HICE, high-intensity continuous exercise; HIIE, high-intensity interval exercise; LV-HIIE, low-volume high-intensity interval exercise; Max-AP, maximum anaerobic power; SIE, sprint interval exercise; VO_2_max, maximal oxygen uptake; All-out: encompasses intensities described by the authors as “sprints”, “maximal”, “maximal exercise”, “exhaustion”, “near the VO_2_max”.

**Table 3:** Individual time-points (TP) of measures of oxidative stress and relevant findings for each study.

Reference	TP0 (0 min)	TP1 (5 min)	TP2 (10 min)	TP3 (20 min)	TP4 (30 min)	TP5 (1 h)	TP6 (2 h)	TP7 (3 h)	TP8 (24 h)	TP9 (48 h)	TP10 (72 h)	Findings
Ammar et al., 2020 [35]	0 min	5 min	10 min	20 min								MDA↑ at TP0 in AnEx; MDA↑ at TP2 in CombEx. MDA continued to increase at TP3 in both Ex; GPX, SOD↑ at TP0 in both Ex; AnEx resulted in greater SOD and GPX at TP0 and TP1. SOD peaked at TP0 in the AnEx; GPX peaked was at TP3 in the CombEx; TAC did not change until TP3 in both Ex.
Baker et al., 2004 [36]	0 min								24 h			MDA↑ at TP0 in the TBM protocol; MDA returned to baseline values at TP8.
Berzosa er al., 2011 [37]	0 min											GPX, CAT and TAC↑ at TP0 in all three Ex; SOD↑ at TP0 in all-out and high intensity continuous Ex.
Bogdanis et al., 2013 [38]					30 min				24 h	48 h		TBARS↑ at TP4 and peaked at TP8 in both pre and post training; Post training resulted in lower TRARS at all TPs. GPX↑ and peaked at TP8 in both pre and post training; CAT↑ and peaked at TP4 in both pre and post training; TAC↑ at TP4 and peaked at TP8 only in pre training. Post training resulted in higher GPX, CAT and TAC in all TPs.
Bouzid et al., 2014 [39]		5 min (MDA)		20 min (SOD, GPX)								MDA↑ at TP1 in old group; GPX and SOD↑ at TP3 in young group.
Djordjevic et al., 2012 [40]	0 min											TBARS↓ at TP0 only in non-athletes; CAT↓ at TP0 only in athletes. GSH did not change in both groups.
Falone et al., 2010 [41]	0 min											MDA did not change at TP0 in both amateur runner and untrained group; TAC↓ at TP0 only in untrained group.
Finkler et al., 2016 [42]		5 min				1 h						TBARS↑ at TP1 and returned to baseline values at TP5; CAT↑ at TP1 and continued to increase at TP5. GPX did not change until TP5.
Fisher et al., 2011 [43]	0 min							3 h	24 h			TBARS↑ at TP0 in the first and second test and at TP7 in the second test; CAT↑ at TP0 in the first and second test; SOD↑ at TP0 and TP7 in all three tests, and returned to baseline value at TP8; GPX↑ at TP0 in the first and third test, and returned to baseline value at TP7.
Groussard et al., 2003 [44]	0 min	5 min	10 min	20 min		40 min						MDA and TBARS↓ at TP3 and TP5; SOD↓ at TP0; GPX did not change until TP5.
Hajizadeh et al., 2017 [45]									24 h			SOD and CAT↑ at TP8 in both HICT and group after 24 weeks’ training; MDA and TAC↑ at TP8 only in HIIE group after 24 weeks’ training.
Jammes et al., 2004 [46]	0 min	5 min	10 min	20 min	30 min							TBARS↑ at TP1 and remained increased until TP4; GSH↓ at TP1 and returned to baseline at TP2.
Jamurtas et al., 2018 [47]	0 min								24 h	48 h	72 h	TBARS and CAT did not change until TP10 in both HIIE and HICE; TAC↑ at TP0 in HICE, and ↑ at TP0 and TP8 in HIIE.
Kyparos et al., 2007 [48]	0 min											TBARS, CAT and TAC↑ at TP0; GSH↓ at TP0.
Miyazaki et al., 2001 [49]	0 min											TBARS↑ at TP0 in both pre- and post-training; Post training resulted in lower TBARS at TP0; SOD, GPX and CAT did not change at TP0.
Parker et al., 2014 [50]	0 min											OS did not change in all three tests; TAC↑ at TP0 in all three test; 100% VO_2_max test resulted in the highest TAC at TP0.
Parker et al., 2018 [51]	0 min					1 h	2 h	3 h				TBARS and SOD↓ at TP0, TP5 and TP6 in both HIIE and SIE; CAT did not change until TP7 in both HIIE and SIE.
Seifi-Skishahr et al., 2008 [52]	0 min						2 h		24 h			MDA↑ at TP6.
Steinberg et al., 2007 [53]	0 min	5 min	10 min	20 min	30 min							TBARS↑ at TP1 and TP2; GSH↓ at TP2 and TP3.
Wadley et al., 2016 [54]	0 min				30 min							TAC↑ at TP4 relative to TP0 in both LV-HIIE and HICE.
Wiecek et al., 2018 [55]		3 min		15 min	30 min	1 h			24 h			SOD↓ at TP3 relative to TP1; SOD↑ at TP4 and returned to baseline value at TP8; CAT↓ at TP3 relative to TP1; CAT↓ at TP5 and returned to baseline value at TP8; GPX↓ at TP3 and ↑ at TP8.

Note: AnEx, Anerobic exercise; CAT, catalase; CombEX, Combined exercise; Ex, exercise; GPX, glutathione peroxidase activity; GSH, glutathione; MDA, malondialdehyde; SOD, superoxide dismutase; TAC, total antioxidant capacity; TBARS, thiobarbituric acid reactive substance; TP, time-point; ↑, significantly increase; ↓, significantly decrease.

**Table 4:** Sociodemographic characteristics of participants.

Reference	Age (Years Old)	Gender	Weight (kg)	BMI	VO_2_max (mL/kg/min)	Diet	Lifestyle	Socio-Economic Level	Tobacco	Alcohol
Ammar et al., 2020 [35]	19.5 ± 1.7	male	71.8 ± 2.1	-	-	no medications and antioxidant dietary supplement	physically inactive	-	-	-
Baker et al., 2004 [36]	23 ± 2	male	75.3 ± 11	-	-	no medications and antioxidant dietary supplement	physically active	university student	-	-
Berzosa et al., 2011 [37]	23 ± 0.41	male	75.25 ± 2.84	23.72 ± 0.69	43.8 ± 1.58	no medications and antioxidant dietary supplement	physically active	-	-	-
Bogdanis et al., 2013 [38]	24.3 ± 1.4	male	77.9 ± 2.9	-	-	no medications and antioxidant dietary supplement	physically active	-	-	-
Bouzid et al., 2014 (young) [39]	20.3 ± 2.8	9 males/6 females	66.1 ± 11.7	-	44.2 ± 5.2	-	sedentary	-	-	-
Bouzid et al., 2014 (old) [39]	65.1 ± 3.57	7 males/8 females	71.8 ± 7.6	23.2 ± 4.4	-	-	sedentary	-	-	-
Djordjevic et al., 2012 (athletes) [40]	17.3 ± 0.2	male	80.9 ± 1.4	23.9 ± 0.3	44.6 ± 0.9	no medications and antioxidant dietary supplement	regular training	-	non-smoking	no alcohol 48 h before test
Djordjevic et al., 2012 (non-athletes) [40]	17.3 ± 0.3	male	81.6 ± 6.1	23.6 ± 1.3	39.7 ± 1.3	no medications and antioxidant dietary supplement	no regular physical activity	-	non-smoking	no alcohol 48 h before test
Falone et al., 2010 (amateur runner) [41]	42 ± 1	male	-	23.5 ± 0.5	48.5 ± 0.9	no medications and antioxidant dietary supplement	regular training	-	-	no alcohol
Falone et al., 2010 (untrained) [41]	39 ± 3	male	-	26.1 ± 1.1	33.3 ± 1.2	no medications and antioxidant dietary supplement	sedentary	no manual labor	-	no alcohol
Finkler et al., 2016 [42]	26.8	male	77.9	23.4	48.9	no medications and antioxidant dietary supplement	physically active	-	non-smoking	-
Fisher et al., 2011 [43]	22 ± 2	male	83 ± 13.6	-	44.6 ± 8.2	no medications and antioxidant dietary supplement	no regular physical activity	-	-	-
Groussard et al., 2003 [44]	22.2 ± 0.6	male	73.4 ± 2.2	-	-	no medications and antioxidant dietary supplement	physically activity	university student	no tobacco in the last 6 months	no alcohol in the last 1 week
Hajizadeh et al., 2017 (HICE) [45]	32.3 ± 7.3	male	81.9 ± 7.2	26.8 ± 5.9	36 ± 4.6	no medications and antioxidant dietary supplement	physically activity	-	no tobacco in the last 6 months	no alcohol in the last 6 months
Hajizadeh et al., 2017 (HIIE) [45]	30.4 ± 8.9	male	83.4 ± 6.3	27.6 ± 4.8	35.9 ± 4.7	no medications and antioxidant dietary supplement	physically activity	-	no tobacco in the last 6 months	no alcohol in the last 6 months
Jammes et al., 2004 [46]	49 ± 3	14 males/5 females	74 ± 3	-	-	-	Sedentary	-	-	-
Jamurtas et al., 2018 [47]	22.4 ± 0.5	male	75.3 ± 8.9	-	45.3 ± 8.4	no medications and antioxidant dietary supplement	-	-	non-smoking	no alcohol in the last 72 h
Kyparos et al., 2007 [48]	21.9 ± 0.9	male	73.9 ± 6.1	-	-	-	-	college student	-	-
Miyazaki et al., 2001 (pre-training) [49]	19.4 ± 0.2	male	70.5 ± 2.6	23.4 ± 0.6	44.9 ± 1.5	-	no regular physical activity	-	-	-
Miyazaki et al., 2001 (post-training) [49]	19.4 ± 0.2	male	70.4 ± 2.7	23.3 ± 0.7	49.7 ± 1.6	-	no regular physical activity	-	-	-
Parker et al., 2014 [50]	22 ± 1	male	81.4 ± 2	25.4 ± 0.7	42.6 ± 2.1	no medications and antioxidant dietary supplement	Sedentary	-	non-smoking	no alcohol in the last 24 h
Parker et al., 2018 [51]	25 ± 2	6 male/2 female	79.4 ± 2.1	25 ± 1	48.4 ± 4	no medications and antioxidant dietary supplement	physically activity	-	non-smoking	no alcohol in the last 24 h
Seifi-Skishahr et al., 2008 [52]	24.1 ± 3.1	-	71.9 ± 9.8	-	34.1 ± 2.7	no medications and antioxidant dietary supplement	Sedentary	-	non-smoking	-
Steinberg et al., 2007 [53]	42 ± 4	9 males/6 females	70 ± 3	22 ± 2	31.7 ± 2.5	-	Sedentary	-	non-smoking	-
Wadley et al., 2016 [54]	22 ± 3	male	-	24 ± 3.1	42.7 ± 5	no medications and antioxidant dietary supplement	-	-	non-smoking	no alcohol in the last 48 h
Wiecek et al., 2018 (female) [55]	22 ± 0.5	female	59.8 ± 2.1	21.5 ± 0.6	-	no medications and antioxidant dietary supplement	physically activity	-	non-smoking	-
Wiecek et al., 2018 (male) [55]	21.6 ± 0.4	male	77.1 ± 2.7	23.7 ± 0.5	-	no medications and antioxidant dietary supplement	physically activity	-	non-smoking	-

**Table 5:** Oxidative stress markers.

Reference	Sample Size	Acute Response on Oxidative Stress and Antioxidant Status							
		MDA	TBARS	OS	TAC	CAT	SOD	GPX	GSH
Ammar et al., 2020 [35] (anerobic)	10	sig ↑	-	-	ns ↑	-	sig ↑ *	sig ↑ *	-
Ammar et al., 2020 [35] (combined)	10	ns ↑	-	-	ns ↑	-	sig ↑ *	sig ↑ *	-
Baker et al., 2004 [36] (TBM)	18	sig ↑ *	-	-	-	-	-	-	-
Baker et al., 2004 [36] (FFM)	18	ns ↑ *	-	-	-	-	-	-	-
Berzosa er al., 2011 [37] (incremental)	34	-	-	-	sig ↑	sig ↑	ns ↑	sig ↑	-
Berzosa er al., 2011 [37] (all-out)	34	-	-	-	sig ↑	sig ↑	sig ↑	sig ↑	-
Berzosa er al., 2011 [37] (70% VO_2_max)	34	-	-	-	sig ↑	sig ↑	sig ↑	sig ↑	-
Bogdanis et al., 2013 [38] (pre-training)	8	-	sig ↑	-	sig ↑	sig ↑	-	ns ↑	-
Bogdanis et al., 2013 [38] (post-training)	8	-	sig ↑	-	ns ↑	sig ↑	-	ns ↑	-
Bouzid et al., 2014 [39] (young)	15	ns ↑ *	-	-	-	-	sig ↑ *	sig ↑	-
Bouzid et al., 2014 [39] (old)	15	sig ↑ *	-	-	-	-	ns ↑ *	ns ↑	-
Djordjevic et al., 2012 [40] (athletes)	58	-	ns ↑	-	-	sig ↓	ns ↓	-	ns ↑
Djordjevic et al., 2012 [40] (non-athletes)	37	-	sig ↓	-	-	ns	ns ↑	-	ns ↑
Falone et al., 2010 [41] (amateur runner)	33	ns ↓ *	-	-	ns *	-	-	-	-
Falone et al., 2010 [41] (untrained)	25	ns ↑ *	-	-	sig ↓ *	-	-	-	-
Finkler et al., 2016 [42]	32	-	sig ↑	-	-	sig ↑	-	ns ↑	-
Fisher et al., 2011 [43] (first)	8	sig ↑	sig ↑	-	-	sig ↑	sig ↑	sig ↑	-
Fisher et al., 2011 [43] (second)	8	sig ↑	sig ↑	-	-	sig ↑	sig ↑	ns ↑	-
Fisher et al., 2011 [43] (third)	8	ns ↑	ns ↑	-	-	ns ↑	sig ↑	sig ↑	-
Groussard et al., 2003 [44]	8	ns ↓	ns ↓	-	-	-	sig ↓	ns	-
Hajizadeh et al., 2017 [45] (HICE)	62	-	-	-	-	-	-	-	-
Hajizadeh et al., 2017 [45] (HIIE)	65	-	-	-	-	-	-	-	-
Jammes et al., 2004 [46]	19	-	ns ↑	-	-	-	-	-	ns ↓
Jamurtas et al., 2018 [47] (HIIE)	12	-	ns ↑	-	sig ↑ *	ns ↑	-	-	-
Jamurtas et al., 2018 [47] (HICE)	12	-	ns ↑	-	sig ↑ *	ns ↑	-	-	-
Kyparos et al., 2007 [48]	11	-	sig ↑	-	sig ↑	sig ↑	-	-	sig ↓
Miyazaki et al., 2001 [49] (pre-training)	9	-	sig ↑ *	-	-	ns	ns ↑	ns ↑	-
Miyazaki et al., 2001 [49] (post-training)	9	-	sig ↑ *	-	-	ns	ns ↑	ns ↓	-
Parker et al., 2014 [50] (70% VO_2_max)	14	-	-	ns ↑	sig ↑ *	-	-	-	-
Parker et al., 2014 [50] (85% VO_2_max)	14	-	-	ns ↑	sig ↑	-	-	-	-
Parker et al., 2014 [50] (100% VO_2_max)	14	-	-	ns ↑	sig ↑ *	-	-	-	-
Parker et al., 2018 [51] (HIIE)	8	-	sig ↓	-	-	ns ↑	sig ↓	-	-
Parker et al., 2018 [51] (SIE)	8	-	sig ↓	-	-	ns ↑	sig ↓	-	-
Seifi-Skishahr et al., 2008 [52]	10	ns ↑	-	-	-	-	-	-	-
Steinberg et al., 2007 [53]	15	ns ↑	-	-	-	-	-	-	ns ↓
Wadley et al., 2016 [54] (LV-HIIE)	10	-	-	-	ns ↓	-	-	-	-
Wadley et al., 2016 [54] (HICE)	10	-	-	-	ns ↓	-	-	-	-
Wiecek et al., 2018 [55]	20	-	-	-	-	ns ↑	ns ↑	ns ↓	-

Note: CAT, catalase; FFM, fat-free mass; GPX, glutathione peroxidase activity; GSH, glutathione; HICE, high-intensity continuous exercise; HIIE, high-intensity interval exercise; LV-HIIE, low-volume high-intensity interval exercise; MDA, malondialdehyde; ns, not significant; SIE, sprint interval exercise; sig, significant; SOD, superoxide dismutase; TAC, total antioxidant capacity; TBARS, thiobarbituric acid reactive substance; TBM, total body mass; VO_2_max, maximal oxygen uptake; *, significant difference between groups.

## Data Availability

The data presented in this study are available on request from the corresponding author. The data are not publicly available due to privacy.

## Appendix A

**Table A1:** Methodological quality assessment questions.

Reporting	
1. Is the hypothesis/aim/objective of the study clearly described?	Yes 1/No 0
2. Are the interventions of interest clearly described? Treatments and placebo (where relevant) that are to be compared should be clearly described.	Yes 1/No 0
3. Are the characteristics of the subjects included in the study clearly described?	Yes 1/No 0
4. Are the main findings of the study clearly described? Simple outcome data (including denominators and numerators) should be reported for all major findings so that the reader can check the major analyses and conclusions.	Yes 1/No 0
5. Have all important adverse events that may be a consequence of the intervention been reported?	Yes 1/No 0
Internal validity—bias	
6. Was an attempt made to blind those measuring the main outcomes of the intervention?	Yes 1/No 0/unable to determine 0
7. Were the statistical tests used to assess the main outcomes appropriate?	Yes 1/No 0/unable to determine 0
Internal validity—confounding (selection bias)	
8. Were study subjects in different intervention groups (trials and cohort studies) or were the cases and controls (case-control studies) recruited over the same period of time?	Yes 1/No 0/unable to determine 0
9. Were study subjects randomized to intervention groups?	Yes 1/No 0/unable to determine 0
Power	
10. Did the study have sufficient power to detect an important effect where the probability value for a difference being due to chance is less than 5%?	Yes 1/No 0/unable to determine 0
Methodological quality assessment questions modified from Downs and Black (1998).	
